# 880. Epidemiology of Burn Injuries Among People Experiencing Homelessness: A Scoping Review

**DOI:** 10.1093/jbcr/irag033.366

**Published:** 2026-04-06

**Authors:** Caitlin M Orton, Geun-woo Oh, Joseph Grobowski, Simrat Kaur, Carly Marincasiu, Haig A Yenikomshian, Julie Hodapp, Barclay T Stewart

**Affiliations:** Department of Surgery, University of Washington Medicine Regional Burn Center, Harborview Medical Center, Seattle, WA; Wayne State University School of Medicine, Detroit, MI; University of Washington Medicine Regional Burn Center, Harborview Medical Center, Seattle, WA; University of Washington Medicine Regional Burn Center, Harborview Medical Center, Seattle, WA; Department of Surgery, University of Washington Medicine Regional Burn Center, Harborview Medical Center, Seattle, WA; Division of Plastic and Reconstructive Surgery, Keck School of Medicine, University of Southern California, Los Angeles, CA; University of Washington, Seattle, WA; Department of Surgery, University of Washington School of Medicine / Harborview Injury Prevention and Research Center, Seattle, WA

## Abstract

**Introduction:**

Homelessness is a rapidly escalating crisis globally. People experiencing homelessness (PEH) face disproportionate burdens of disability, illness and addiction, compounded by limited resource access and daily exposure to violence and extreme weather. These intersecting vulnerabilities increase their risk for burn injury and complicate already challenging recoveries. This scoping review examines the current state of knowledge on burn injuries among PEH and contrasts their epidemiology with domiciled patients (DP).

**Methods:**

We conducted a scoping review guided by Arksey and O’Malley’s methodological framework and reported according to the PRISMA Extension for Scoping Reviews. With librarian support, systematic searches were performed in PubMed, Embase, Cumulative Index to Nursing and Allied Health Literature, Academic Search Complete, Web of Science Core Collection, ProQuest Social Services Abstracts and ProQuest Dissertations & Theses Citation Index. A narrative synthesis was completed to characterize the risks and incidence of burn injuries among PEH, describe their injury patterns, recovery and rehabilitation, and compare these with DP. Reports were organized by geographic region and generalizability of findings.

**Results:**

Thirty-six reports were analyzed (26 peer-reviewed manuscripts, 9 abstracts, 1 thesis). Twenty-two reports were from West Coast (WC) burn centers. Within the US, PEH were more often male and of White and Black race, and more frequently presented with substance misuse and mental illness histories compared to DP. PEH were more likely to have sustained injuries from fire, assault and self-immolation. WC data suggested larger burn sizes among PEH, whereas national and other regional data reported no differences. Reports from national registries identified higher rates of full-thickness burns in PEH, while WC studies did not. PEH had longer hospitalizations, amassed higher charges, were more likely to leave against medical advice and less likely to receive follow-up care. No consistent differences were found in rates of inhalation injury, inpatient complications, mortality, or amputations.

**Conclusions:**

Understanding the unique injury epidemiology and recovery of PEH is critical to designing effective prevention strategies and improving burn care. These disparities highlight the need for integrated trauma-informed care models that address both burn management and the social and behavioral health challenges PEH encounter.

**Applicability of Research to Practice:**

PEH face unique and heightened risks for burn injury, demanding prevention strategies that are grounded in the realities of their daily lives. Effective care for PEH with burn injuries requires coordinated, trauma-informed, multidisciplinary teams and partnerships with community organizations to bridge hospital and community care, build trust and promote recovery.

**Funding for the study:**

The contents of this abstract were developed under a grant from the National Institute on Disability, Independent Living, and Rehabilitation Research (NIDILRR grant #90DPBU0005).

